# Accumulation of α-synuclein in dementia with Lewy bodies is associated with decline in the α-synuclein-degrading enzymes kallikrein-6 and calpain-1

**DOI:** 10.1186/s40478-014-0164-0

**Published:** 2014-12-05

**Authors:** J Scott Miners, Ruth Renfrew, Marta Swirski, Seth Love

**Affiliations:** Dementia Research Group, University of Bristol, Level 1, Learning and Research, Southmead Hospital, BS10 5NB Bristol, UK

**Keywords:** Dementia with lewy bodies, Calpain-1, Kallikrein-6, α-synuclein

## Abstract

Kallikrein-6 and calpain-1 are amongst a small group of proteases that degrade α-synuclein. We have explored the possibility that reduction in the level or activity of these enzymes contributes to the accumulation of α-synuclein in Lewy body diseases. We measured calpain-1 activity by fluorogenic activity assay, kallikrein-6 level by sandwich ELISA, and levels of α-synuclein and α-synuclein phosphorylated at serine 129 (α-synuclein-P129), in post-mortem brain tissue in pure dementia with Lewy bodies (DLB, n = 12), Alzheimer’s disease (AD, n = 20) and age-matched controls (n = 19). Calpain-1 activity was significantly reduced in DLB within the cingulate and parahippocampal cortex, regions with highest α-synuclein and α-synuclein-P129 load, and correlated inversely with the levels of α-synuclein and α-synuclein-P129. Calpain-1 was unaltered in the thalamus and frontal cortex, regions with less α-synuclein pathology. Kallikrein-6 level was reduced in the cingulate cortex in the DLB cohort, and correlated inversely with α-synuclein and α-synuclein-P129. Kallikrein-6 was also reduced in DLB in the thalamus but not in relation to α-synuclein or α-synuclein-P129 load and was unaltered in the frontal and parahippocampal cortex. In SH-SY5Y cells overexpressing wild-type α-synuclein there was partial co-localisation of kallikrein-6 and calpain-1 with α-synuclein, and siRNA-mediated knock-down of kallikrein-6 and calpain-1 increased the amount of α-synuclein in cell lysates. Our results indicate that reductions in kallikrein-6 and calpain-1 may contribute to the accumulation of α-synuclein in DLB.

**Electronic supplementary material:**

The online version of this article (doi:10.1186/s40478-014-0164-0) contains supplementary material, which is available to authorized users.

## Introduction

Abnormal aggregation of α-synuclein (α-syn) within neuronal perikarya (Lewy bodies) and neurites (Lewy neurites) are defining neuropathological hallmarks of Parkinson’s disease (PD) and dementia with Lewy bodies (DLB). There is accumulation of both insoluble [1] and soluble oligomeric α-syn [2] in sporadic PD and DLB without an increase in α-syn mRNA [3,4], suggesting a major role for impaired clearance in the pathogenesis of PD and DLB. Several genetic mutations that cause PD (e.g. in *PINK1*, *PARK2*, *LRRK2*) involve proteins associated with the ubiquitin-proteasome system (UPS) and the autophagy-lysosomal pathway (ALP), which have established roles in α-syn clearance (reviewed in [5-8]) and are dysregulated in sporadic PD and DLB. Cathepsin D (CTSD) the major lysosomal protease responsible for α-syn cleavage [9-11] is upregulated in PD and DLB [12]. A small number of non-lysosomal proteolytic enzymes have been identified that cleave α-syn *in vitro*, including MPP-3, −9 [13], calpain-1 (CAPN1) [14,15] and kallikrein-6 (KLK6) [16,17]. These are expressed within the cytosol, plasma membrane or are secreted extracellularly.

KLK6 (also known as neurosin) is a serine protease predominantly expressed within the brain, especially in neurons and oligodendrocytes [18-20]. *In vitro*, KLK6 degrades monomeric α-syn within the NAC region, essential for filament assembly, and protects against α-syn aggregation and neurotoxicity [16,17]. KLK6 co-localises with α-syn in Lewy bodies in post-mortem brain tissue in PD [16,21]. Direct interaction between KLK6 and α-syn in mouse brain was demonstrated by co-immunoprecipitation [16]. The authors also showed that KLK6 cleaved α-syn when the enzyme was released from mitochondria under stress conditions. KLK6 is also secreted in an active form extracellularly and degrades extracellular α-syn [22]; this may be relevant in preventing the transcellular spread of α-syn that is thought to be important in the propagation of disease [23,24]. Phosphorylated forms of α-syn which are more abundant in Lewy body diseases, and mutant forms of α-syn that accumulate in familial forms of PD, are resistant to proteolytic cleavage by KLK6 [17]. A recent study showed that elevating the level of KLK6 *in vivo* protected against α-syn aggregation and toxicity in a rat model of PD [25] and that KLK6 level was significantly lower in mid-temporal cortex obtained post mortem from 8 patients with DLB than from 6 controls.

Calpains are calcium-dependant cysteine proteases involved in multiple and diverse cellular processes. Calpain-1 (CAPN1, μ-calpain) and calpain-2 (CAPN2, m-calpain) are regulated by micromolar and millimolar levels of intracellular calcium, respectively, and unlike other members of the calpain family are highly expressed within the CNS. CAPN1 and CAPN2 are dysregulated in AD and other tauopathies [26,27]. CAPN1 cleaves α-syn *in vitro* [14,15]. It is neuronally expressed and likely to interact with α-syn within the pre-synaptic terminal [28]. However, to our knowledge its activity in Lewy body diseases has not been studied. CAPN1 cleaves monomeric α-syn within the NAC region. This would be predicted to prevent aggregation and neurotoxicity, and indeed the addition of CAPN-1 cleavage fragments of monomeric α-syn inhibited aggregation of the full-length monomers [15]. However, cleavage of fibrillar rather than monomeric α-syn by CAPN1 promoted the aggregation of full-length α-syn [14,29] and might therefore be expected to accelerate disease progression.

In this study, we investigated whether KLK6 level and CAPN1 activity differed significantly between pure DLB, AD and age-matched controls, in 4 grey matter regions that vary in their predilection to Lewy body pathology: parahippocampal, cingulate and frontal cortex, and posterior thalamus. After establishing that KLK6 level and CAPN1 activity were significantly reduced in cingulate cortex in DLB, we showed that the reductions correlated inversely with the levels of total α-syn and α-syn phosphorylated at serine-129 (α-syn-P129). To assess the functional relevance of down-regulation of KLK6 level and CAPN1, we used SH-SY5Y neuroblastoma cells, within which there was partial co-localisation of KLK6 and CAPN1 with α-syn. Reduction of KLK6 and CAPN1 expression by siRNA-mediated knock-down elevated endogenous α-syn level within these cells. Together, our findings suggest that reductions in KLK6 and CAPN1 contribute to the accumulation of α-syn in sporadic DLB.

## Materials and methods

### Study cohort

Brain tissue was obtained from the South West Dementia Brain Bank, University of Bristol. The study had local Research Ethics Committee approval. We examined DLB (n = 12) and controls (n = 19) that were matched for age, gender and post-mortem (PM) delay (Table 1, and the MRC UK Brain Banks Network database identifiers are listed in Additional file 1: Table S1). For further comparison, we also included an AD cohort (n = 20). The brains had been separated midsagittally - the left half sliced and frozen at −80°C and the right half fixed in formalin for paraffin histology and detailed neuropathological examination. All disease cases were diagnosed using widely accepted neuropathological criteria [30]. Pure DLB was diagnosed according to consensus neuropathological criteria [31]. All DLB cases had Braak stage 5 or 6 Lewy body pathology [32]. Controls had no history of dementia and no neuropathological abnormalities apart from mild neurofibrillary tangle pathology (Braak tangle stage III or less) [33] and scattered diffuse Aβ plaques in some cases. No Lewy body pathology was present in the substantia nigra or cerebral cortex in either the AD cases or the controls.

**Table 1:** **Cases studied** ^**a**^

	**Age-at-death (y)**	**Gender (M:F)**	**Post-mortem delay (h)**
**Control (n = 19)**	80.3 ± 9.0^b^	14:5	37.4 ± 14.3^b^
**DLB (n = 12)**	76.8 ± 9.1	7:5	27.7 ± 9.1
**AD (n = 20)**	79.4 ± 7.3	8:12	28.2 ± 16.3

^a^For a full list of all cases with MRC brain bank identifiers please see Additional file 1: Table S1.

^b^Mean ± SD.

### Immunohistochemical assessment of α-syn and α-syn-P129

Formalin-fixed paraffin-embedded sections of frontal, cingulate, parahippocampal cortex and thalamus from all DLB cases were immunolabeled for α-syn (80 μg/ml; Vector Labs, Peterborough, UK) and α-syn-P129 (0.8 μg/ml; Abcam) by means of a standard streptavidin-biotin-HRP immunohistochemistry protocol [34]. The extent of immunolabeling of each antigen was measured by field fraction analysis with the help of Image Pro Plus™ software (Media Cybernetics, Marlow**,** UK) driving a Leica DM microscope with a motorized stage. The software made an unbiased selection of twelve x20-objective fields and the percentage area immunopositive for the relevant antigen was determined for each section, as outlined previously [35,36].

### Tissue preparation

Brain tissue was dissected from 4 cortical areas: parahippocampal cortex (Brodmann area 36), cingulate cortex (Brodmann area 24), frontal cortex (Brodmann area 8) and posterior thalamus (pulvinar). Brain tissue (200 mg) samples were sequentially extracted in 1% NP-40 buffer (140 mM NaCl, 3 mM KCl, 25 mM Tris ph 7.4, 5 mM EDTA, 2 mM 1,10-phenanthroline, 0.1 M PMSF, 1.7 mg/ml aprotinin, NP-40 detergent, 100 ml dH_2_O) (soluble extract) in a Precellys 24 homogeniser (Stretton Scientific, Derbyshire, UK) with 2.3 mm ceramic beads (Biospec, Glasgow, UK). The homogenates were spun at 13000 g for 15 min at 4°C and the supernatant was removed and stored at −80°C. Insoluble material was solubilized by vigorous agitation in 6 M GuHCl, re-homogenized and left for 4 h at room temperature (RT) before storage at −80°C.Total protein concentrations were determined using the Total Protein Kit (Sigma Aldrich, Dorset, UK) following the manufacturer’s guidelines.

### CAPN1 fluorogenic activity assay

CAPN1 activity was measured in NP-40 soluble brain tissue homogenates using an internally quenched fluorogenic substrate for CAPN1 derived from the CAPN1-specific cleavage site in α-spectrin (1 μM, H-K(FAM)-EYY ~ GMMK(DABCYL)-OH (Millipore)) in the presence of a calpain-1 specific inhibitor (10 μM, Tocris, Bristol, UK). Brain tissue homogenates (10 μl diluted in 40 μl assay buffer) and purified human CAPN1 (Sigma Aldrich, Dorset, UK) were diluted in assay buffer (50 mM Tris–HCl, 50 mM NaCl, 5 mM β-mercaptoethanol, 5 mM CaCl_2_, 1 mM EGTA, 1 mM EDTA) pre-warmed to 37°C as optimised by Mittoo et al. [37]. Each sample and standard was added to duplicate wells, with or without inhibitor and left for 10 min at 37°C, prior to addition of the fluorogenic substrate which was also diluted in assay buffer. The reaction was left to proceed at 37°C for 3 h in the dark after which fluorescence was read using a FLUOstar Optima plate reader with excitation at 490 nm and emission at 518 nm. For each sample the fluorescent signal in the inhibited wells was subtracted from that in the uninhibited wells. Each sample was measured in duplicate and measurements were repeated on two separate occasions. Calpain-1 activity was interpolated from measurements on serial dilutions of calpain-1 (20–1.25 μg/ml) that were included in each plate.

### KLK6 sandwich ELISA

KLK6 level was determined in NP-40 soluble brain tissue homogenates using a sandwich ELISA that was developed in-house. Mouse monoclonal anti-KLK6 antibody (Sigma Aldrich, Dorset, UK) diluted in PBS (1:200) left overnight at room temperature (RT) in the wells of clear Costar™ high-binding 96-well microplates (R&D systems, Abingdon, UK). The plates were washed in PBS 0.01% tween-20 and blocked for 3 h in 1%BSA/PBS at 26°C. Tissue homogenates (6.5 μg total protein) and human recombinant standards (R&D systems, UK) diluted in 1% BSA/PBS were added overnight at 4°C. After another 5 washes, biotinylated goat polyclonal anti-KLK6 antibody (1 μg/ml) (R&D systems, Cambridge, UK) diluted in 1% BSA/PBS, was added for 2 h at 26°C. After washing, streptavidin:HRP (1:200) (R&D systems, UK) diluted in PBS/0.01% tween-20 was added for 1 h at 26°C, washed, and chromogenic substrate (TMBS, R&D systems, UK) was added for 20 min in the dark. The reaction was stopped with 2 N sulphuric acid and absorbance read at 405 nM in a FLUOstar Optima plate reader. KLK6 level was determined by interpolation against measurements on serial dilutions of recombinant human KLK6 (720–11.25 ng/ml). All sample measurements fell within the calibration curve and measurements for each sample were made in duplicate.

### α-syn sandwich ELISA

α-syn level was determined in GuHCl solubilised brain extracts by sandwich ELISA, as previously described [38,39]. Mouse monoclonal anti-α-syn antibody (Syn-1, raised against epitope corresponding to aa 91–99 [40]) (0.5 μg/ml) (BD Biosciences, Oxford, UK) was coated onto a NUNC maxisorp 96-well plate overnight at RT. The plate was washed in PBS/0.01% tween-20 and blocked for 1.5 h in 1% BSA/PBS. Tissue samples diluted in PBS were added for 2 h at RT with constant shaking. Following a further wash step, biotinylated goat polyclonal anti-α-syn (raised against full-length recombinant human α-syn) (1 μg/ml) (R&D systems, UK) was added for 2 h at RT. After washing, streptavidin:HRP (1:200) (R&D systems) was added for 20 min, the plates washed, and chromogenic substrate (TMBS, R&D system) was added for 20 min in the dark. The reaction was stopped with 2 N sulphuric acid and absorbance at 405 nM read in a FLUOstar Optima plate reader. Total α-syn level was determined by interpolation against readings on serial dilutions of recombinant human α-syn (62.5-0.97 ng/ml) (rPeptide, Stratech, Suffolk, UK) on each plate. Measurements for each sample were made in duplicate.

### α-syn-P129 sandwich ELISA

α-syn-P129 level was determined in GuHCl solubilised brain extracts by sandwich ELISA as previously described [38,39]. Mouse monoclonal anti-α-syn antibody (0.5 μg/ml) (BD Biosciences, Oxford, UK) was coated onto a NUNC maxisorp 96-well plate overnight at RT. The plate was washed in PBS/0.01% tween-20 and blocked for 1.5 h in 1% BSA/PBS. Tissue samples (1 μl plus 99 μl PBS) were added for 5 h at RT with constant shaking. After washing, biotinylated anti-α-syn-129 (0.8 μg/ml) (Abcam, Cambridge, UK) was added at 4°C overnight. Following washing, biotinylated horse anti-rabbit antibody (Vector Labs, Burlingame, CA, USA) diluted 1:1000 in PBS with 0.01% tween-20 was added for 1 h at RT. After washing, streptavidin:HRP (1:200) (R&D systems) was added for 20 min, the plates washed and chromogenic substrate (TMBS, R&D system) was added for 20 min in the dark. The reaction was stopped with 2 N sulphuric acid and absorbance at 405 nM read in a FLUOstar Optima plate reader. α-syn-P129 was determined by interpolation against readings on serial dilutions (200–3.25 ng/ml) of recombinant α-syn that had been phosphorylated at serine 129 by incubation with casein kinase II (New England Biolabs, Hitchin, UK) for 1 h at 30°C in the presence of ATP (New England Biolabs).

The specificity of the α-syn-P129 detection antibody used in the α-syn-P129 sandwich ELISA has previously been confirmed by dot blot, as described in [39].

### SH-SY5Y cell culture

SH-SY5Y neuroblastoma cells stably expressing human full-length α-syn (SH-SY5Y-α-syn), were provided by Dr Rohan de Silva, University College London (UCL), UK and were generated by transfection with a pCDNA3.1 vector (Life Technologies, UK) containing wild-type human *SNCA* cDNA under the control of a CMV promoter, as described [38]. Transfection was carried out with TransFast (Promega) followed by selection of clones in culture medium containing 0.3 mg/ml G418 (Geneticin, Life Technologies). Stable clones were further maintained in culture containing G418. SH-SY5Y-α-syn cells were routinely maintained in 42% vol/vol Ham’s F12 nutrient mixture (F12) (Sigma, UK), 42% vol/vol Eagle’s minimum essential medium (MEM) (Sigma, UK), 15% vol/vol foetal calf serum (FCS) (Sigma, UK), 2 mM l-glutamine (Sigma, UK), 1% vol/vol non-essential amino acids (NEAA) solution (Sigma, UK), 20 units/mL penicillin/20 mg/mL streptomycin (Sigma, UK) and 250 ng/mL amphotericin B (Life Technologies, UK) at 37°C in 5% CO_2_, 21% O_2_. SH-SY5Y-α-syn cells were maintained under constant selection pressure by use of 0.3 mg/ml G418 (Geneticin, Life Technologies).

### siRNA knock-down of KLK6 and CAPN1

For siRNA-mediated transfection, SH-SY5Y-α-syn cells were grown to 30-50% confluence. KLK6 and CAPN1 siRNA (10 nM) (Santa Cruz, Dallas, Texas, U.S.A) diluted in Optimum™ (Invitrogen, Paisley, UK) was conjugated to INTERFERin (Polyplus Transfection, Illkirch, France) according to the manufacturer’s guidelines and added to the cells. The culture medium was exchanged for fresh complete medium after 4 h to minimise cellular toxicity. After 4 d the cells were washed in PBS, detached in PBS without magnesium and chloride (Sigma Aldrich, Dorset, UK), pelleted and lysed in non-denaturing CellLyticTM cell lysis buffer according to manufacturer’s guidelines (Sigma Aldrich, Dorset, UK). The samples were centrifuged at 13,000 rpm for 15 min at 4°C and the supernatants aliquoted and stored at −80°C until used. Total protein level was determined using Total Protein Kit (Sigma Aldrich, Dorset, UK) and an equal amount of total protein from each sample was added for measurement of KLK6 level, CAPN1 activity and α-syn levels by sandwich ELISA or activity assay, as described above.

### Double immunofluorescent labeling of CAPN1/KLK6 and α-syn

SH-SY5Y-α-syn cells were grown on poly-L-lysine coated coverslips, washed in PBS x3, fixed in 4% paraformaldehyde for 10 min, further washed in PBS and blocked for 30 min in PBS with 0.01% triton-X100 and 5% horse serum (Millipore, Beeston, Nottingham, UK). Goat polyclonal anti-KLK6 (2 μg/ml) (R&D systems, UK) or rabbit polyclonal anti-CAPN1 (10 μg/ml) (BIOSS, Woburn, Massachusetts, MA, USA) was added together with mouse monoclonal α-syn (2.5 μg/ml) (BD, Oxford, UK) for 1 h at RT. Alexa-fluor-488 and −555 (Invitrogen, Life Technologies, Paisley, UK) were then added at 1:1000 for 1 h at RT in the dark. Images were acquired under a x100 objective with a confocal scanning laser microscope.

### Statistical analysis

Whenever possible, parametric statistical tests were used for comparisons between groups (in some cases this required logarithmic transformation of the data to obtain a normal distribution). ANOVA with Bonferroni post-hoc test was used for multiple-group comparisons. For variables that were not normally distributed even after transformation, Kruskall-Wallis test was used, with Dunn’s test for pairwise intergroup comparisons. Pearson analysis was used to assess the correlation between pairs of variables. Statistical tests were performed using SPSS version 16. P-values < 0.05 were considered statistically significant.

## Results

### CAPN1 activity is reduced in DLB and correlates inversely with α-syn in regions affected by Lewy body pathology

**Figure 1:**
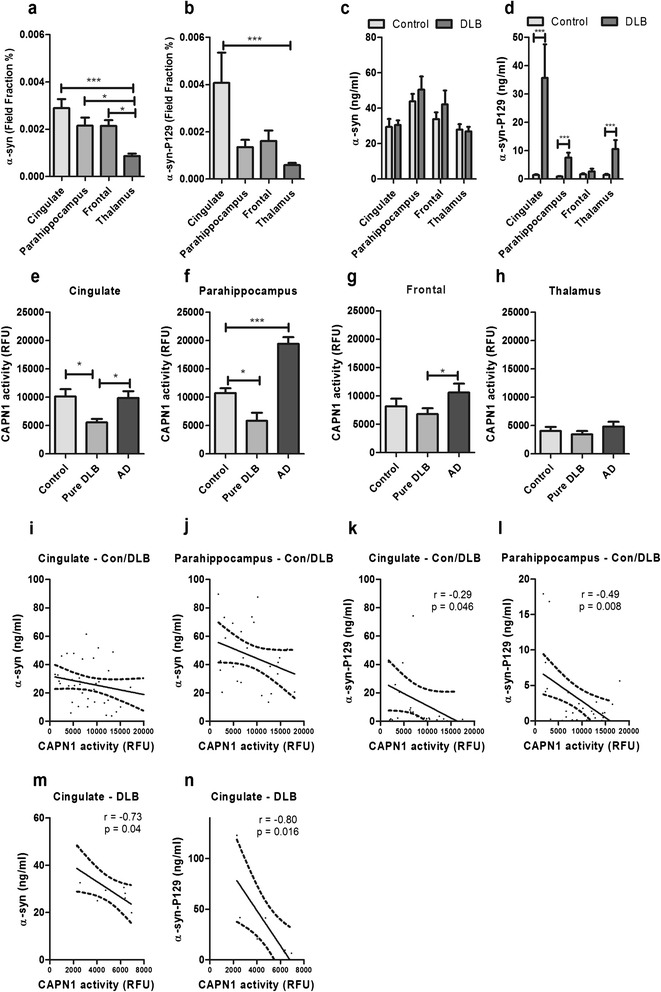
**Regional, disease-specific reduction in CAPN1 activity in DLB, and inverse correlation with α-syn load in human post-mortem brain tissue. a** α-syn and **b** α-syn-P129 immunopositive field fractions in DLB (there was no labelling in controls), and **c** α-syn and **d** α-syn-P129 levels in DLB and controls as measured by sandwich ELISA in brain tissue extracts, varied in a regional dependent-manner. The field fraction and level of α-syn-P129 were highest in the cingulate cortex. The α-syn field fraction was also highest in the cingulate cortex (and lowest in the thalamus). CAPN1 activity was significantly reduced in DLB in the **e** cingulate and **f** parahippocampal cortex but not in **g** frontal cortex or **h** thalamus. CAPN1 activity was increased in the **f** parahippocampal gyrus in AD but not in other regions. In a combined cohort, CAPN1 activity tended to correlate inversely with the levels of **i-j** α-syn, although this did not reach significance and significantly with **k-l** α-syn-P129 in the cingulate and parahippocampal cortex. In the cingulate cortex, CAPN1 activity correlated inversely with **m** α-syn and **n** α-syn-P129 in the DLB group alone. In **a-h** the horizontal bars indicate the mean value and SEM. In **i-n** the solid lines indicate best fit linear regression and the interrupted lines the 95% confidence intervals. In all of the panels, each point represents a separate brain. *P < 0.05 ***P < 0.001.

We initially assessed α-syn and α-syn-P129 load within the DLB cohort by standard immunoperoxidase labelling (Figure 1a-b) and within brain extracts (extracted in GuHCl) by sandwich ELISA in both control and DLB cohorts (Figure 1c-d). Immunolabeling of α-syn (p = 0.004) (Figure 1a) and α-syn-P129 (p = 0.0022) (Figure 1b) varied significantly between regions and was highest in cingulate cortex > parahippocampal cortex > frontal cortex > thalamus. α-Syn level, measured by sandwich ELISA, did not vary significantly between DLB and controls in any of the regions (Figure 1c). However, α-syn-P129 was markedly elevated in the cingulate cortex compared to other regions, and α-syn-P129 level was higher in DLB than controls in all regions, except for the frontal cortex (Figure 1d).

We next examined CAPN1 activity in the same brain extracts for which we had measured α-syn and α-syn-P129 in the DLB and control cohorts, in all four regions. CAPN1 was significantly lower in pure DLB than age-matched controls in the cingulate cortex (Figure 1e) and parahippocampal cortex (Figure 1f). CAPN1 activity was not reduced in frontal cortex (Figure 1g) or thalamus (Figure 1h). CAPN1 activity was significantly increased in the parahippocampal cortex in AD (Figure 1f). However, CAPN1 activity in AD brains was similar to that in control brains in all other regions.

When the DLB and control cohorts were combined for analysis a trend towards an inverse correlation was observed between CAPN1 activity and α-syn in the cingulate (Figure 1i) and parahippocampal cortex (Figure 1j) but did not reach statistical significance. CAPN1 activity was significantly correlated inversely with α-syn-P129 in the cingulate (Figure 1k) and parahippocampus (Figure 1l). In DLB cases alone, CAPN1 correlated inversely with α-syn (Figure 1m) and α-syn-P129 (Figure 1n) in the cingulate cortex only. Correlations were not observed in the frontal cortex or thalamus (data not shown).

### KLK6 level is reduced in DLB and correlates inversely with α-syn in the cingulate cortex

**Figure 2:**
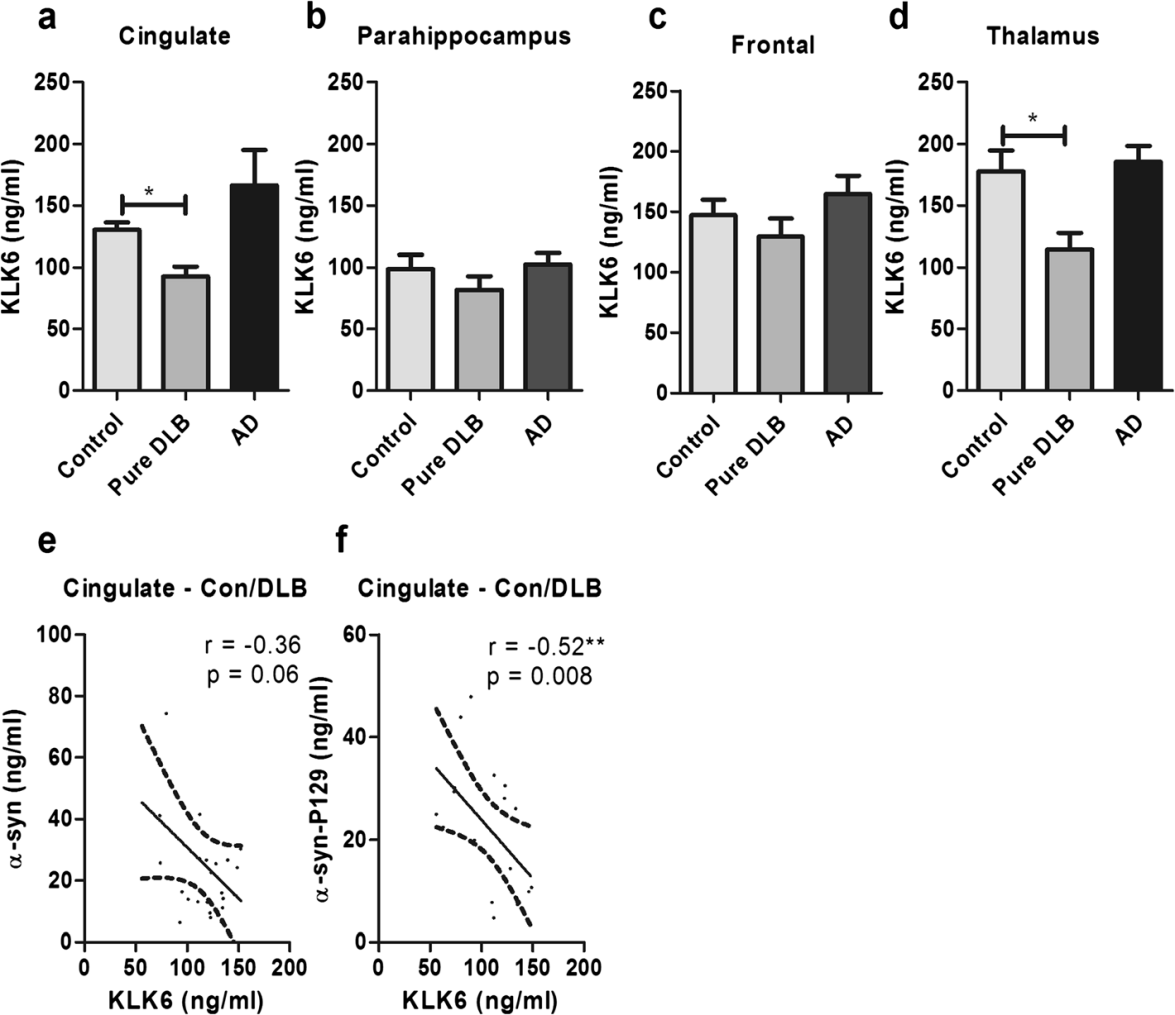
**Regional, disease-specific reduction in KLK6 level in DLB, and inverse correlation with α-syn load in human post-mortem brain tissue.** KLK6 level was reduced significantly in the **a** cingulate cortex and **d** thalamus in DLB but not in the **b** parahippocampal cortex or **c** frontal cortex. In the cingulate cortex only, KLK6 correlated inversely with **e** total α-syn and **f** α-syn-P129 level. In **a-d** the horizontal bars indicate the mean value and SEM. In **e-f** the solid lines indicate best fit linear regression and the interrupted lines the 95% confidence intervals. In all of the panels, each point represents a separate brain. *P < 0.05.

KLK6 level was significantly lower in pure DLB than in age-matched controls in the cingulate cortex (Figure 2a) and thalamus (Figure 2d). KLK6 was unaltered in the frontal and parahippocampal cortex (Figure 2b-c). When the DLB and control cohorts were combined for analysis, an inverse correlation was observed between KLK6 level and α-syn in the cingulate cortex that approached significance (p = 0.06) (Figure 2e). A significant negative correlation was observed between KLK6 level and α-syn-P129 (Figure 2f) in the cingulate cortex but not in any other region.

### CAPN1 and KLK6 co-localise with α-syn in vitro

**Figure 3:**
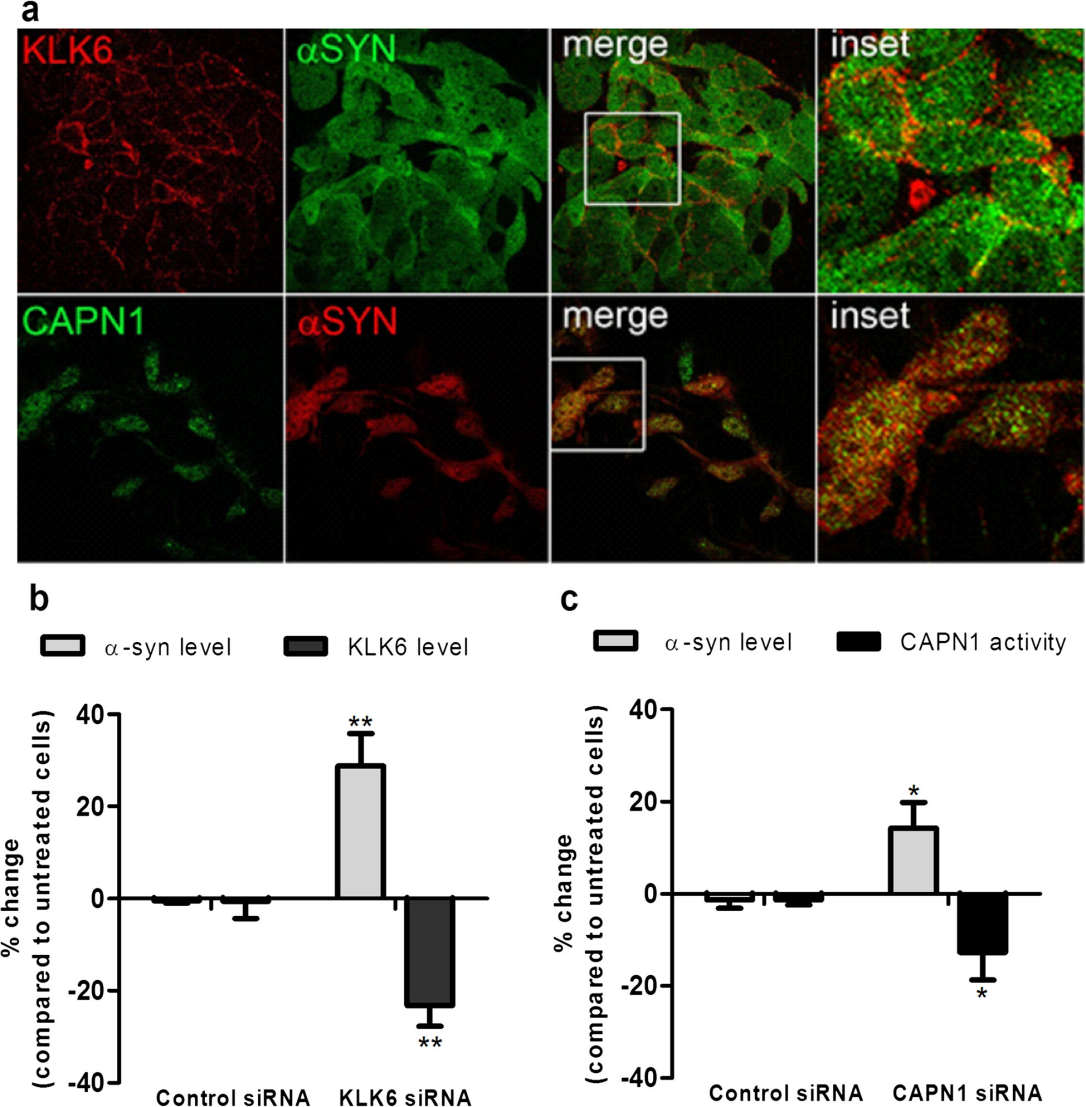
**KLK6 and CAPN1 are partially co-localised with α-syn and regulate its level in human SH-SY5Y neuroblastoma cells. a** Double-labelling immunofluorescence and confocal microscopy shows partial co-localisation of KLK6 and CAPN1 with α-syn. KLK6 (red) has a distinctive distribution pattern, along the plasma membrane, where it co-localises with some of the α-syn (top merged image and the magnified inset image). CAPN1 (green) has a widespread punctate cytosolic distribution and co-localises with α-syn within the cytoplasm (bottom merged image and in the magnified inset image). **b-c** siRNA-mediated knock-down of KLK6 or CAPN1 in SH-SY5Y cells overexpressing α-syn significantly increased endogenous α-syn level. The experiments were repeated in triplicate. The error bars represent the SEM. *P < 0.05 **P < 0.01.

To explore further the interaction between α-syn and the two enzymes, CAPN1 and KLK6, we used human SH-SY5Y cells transfected with full-length human *SNCA*. SH-SY5Y cells overexpressing wild-type α-syn (SH-SY5Y-α-syn) had a widespread, predominantly cytosolic distribution of α-syn (Figure 3a). KLK6 had a distinctive distribution pattern, predominantly along the plasma membrane, and co-localised with a minor fraction of endogenous α-syn (Figure 3a, upper panels). CAPN1 had a more widespread punctate cytosolic distribution and co-localised with α-syn within the cytoplasm (Figure 3a, lower panels).

### Reduction in CAPN1 or KLK6 causes an increase in endogenous α-syn

To determine the functional significance of a potential interaction between KLK6 or CAPN1 and α-syn, we examined the effect on α-syn level of siRNA mediated knock-down of KLK6 or CAPN1 in SH-SY5Y-α-syn cells. A small reduction in the expression of either enzyme resulted in a significant increase in endogenous α-syn. A 20% decrease in KLK6 level produced a ~25% increase in α-syn (P < 0.01) (Figure 3b) and a 15% reduction in CAPN1 activity resulted in a ~15% increase in α-syn (P < 0.05) (Figure 3c).

## Discussion

We have shown that (i) KLK6 and CAPN1 are reduced in post-mortem brain tissue in DLB, in regions of cerebral cortex that show most marked accumulation of α-syn, (ii) the amount of α-syn in these regions correlates inversely with KLK6 and CAPN1 level and activity, (iii) KLK6 and CAPN1 partially co-localised with α-syn in SH-SY5Y cells overexpressing wild-type α-syn, and (iv) the level of α-syn within these cells increases after down-regulation of KLK6 and CAPN1 by siRNA knock-down. The present findings suggest that impaired proteolytic clearance by KLK6 and CAPN1 could contribute to the accumulation of α-syn in DLB.

CAPN1 is expressed abundantly in neurons and is enriched in pre-synaptic terminals where it may interact physiologically with α-syn [28]. We have shown that CAPN1 also co-localises with α-syn within the cytoplasm of human SH-SY5Y cells, and have confirmed a previous report [11] that siRNA-mediated reduction in *CAPN1* results in a significant increase in endogenous wild-type α-syn. In human cingulate and parahippocampal cortex, regions with a predilection for α-syn pathology, we demonstrated a ~50% reduction in CAPN1 activity in patients with DLB. CAPN1 cleavage of monomeric α-syn has been fully characterized [14,15,29] and the major cleavage site located between amino acids 83–84 [14] is located within the NAC region (aa 61–95) which is required for α-syn aggregation [41,42]. Cleavage of α-syn by CAPN1 was shown to inhibit aggregation of full-length α-syn [15]. Phosphorylation at serine-129 is the predominant modification of α-syn in Lewy bodies and Lewy neurites [43,44] and has been reported to mediate both the aggregation and toxicity of α-syn [45-47]. A reduction in CAPN1 activity in cingulate and parahippocampal cortex would therefore be expected to cause aggregation and accumulation of α-syn and, indeed, we found that α-syn-P-129 levels correlated inversely with CAPN1 activity. The reduction in CAPN1 activity was regionally specific (it was not observed in frontal cortex or thalamus) and was also disease-specific in that a reduction was not observed in AD.

Although these data may predict a role for reduced CAPN1-mediated cleavage of α-syn in the pathogenesis of Lewy body diseases, some questions remain. Firstly, CAPN1 cleaves fibrillar, as well as monomeric α-syn, to produce C-terminal fragments which promote the aggregation and neurotoxicity of full-length α-syn [14,15,29]. Thus CAPN1 may prevent the formation of Lewy bodies from soluble monomeric α-syn but promote the aggregation and toxicity of aggregated species of α-syn. This may explain why CAPN-specific inhibitors ameliorated neuronal and behavioural abnormalities in an experimental mouse model of PD in which CAPN1 level was elevated [48]. A recent study showed that overexpression of the endogenous CAPN1 inhibitor, calpastatin, resulting in reduced calpain activity, reduced truncated and aggregated α-syn and synaptic impairment in a mouse model of PD [49]. Secondly, CAPN1 expression was also reportedly elevated in post-mortem brain tissue in PD [48,50,51], in contrast to our findings in DLB, although these studies relied upon semi-quantitative immunohistochemical assessment or measurement of spectrin rather than CAPN1 itself and did not measure enzyme activity. The regions sampled might also account for these disease-specific differences; for instance, the cortical regions we sampled in DLB are mostly preserved compared to extensive neurodegeneration in subcortical areas in PD, such as the substantia nigra. Thirdly, we have not fully characterized the levels of CAPN-1 specific cleavage fragments in our brain extracts, an approach used successfully in other studies [14,15,29], or addressed whether CAPN1 specific substrates, such as α-spectrin, vary in relation to CAPN1 activity.

Lastly, attempts should be made to address whether reduction in CAPN1 activity in DLB in areas with significant pathology represents a failed compensatory response to increasing α-syn or occurs as a consequence of disease. The former might explain why CAPN1 activities are lowest in the thalamus, a region without significant pathology. Alternatively, albeit less likely, it is possible that individuals with lower CAPN1 activities are at greater risk of developing DLB. In further studies of the possible role of reduced CAPN1 activity in DLB it will be important to consider the roles of CAPN1 in addition to its potential for cleavage of α-syn. For instance, CAPN1 has been localized intracellularly and has been implicated in the dysregulation of autophagy [52-54] – implicated in both PD and DLB.

KLK6 is abundantly expressed within the brain, particularly in neurons and oligodendrocytes [18-20]. It degrades α-syn *in vitro* [16] and co-localises with Lewy bodies in post-mortem brain tissue from people with PD [16,21]. Iwata A et al. [16] demonstrated an interaction between KLK6 and α-syn in mouse brain by co-immunoprecipitation and double-immunofluorescence. We have shown that KLK6 is partly co-localised with α-syn in SH-SY5Y neuroblastoma cells, in a distinctive subplasmalemmal distribution, and confirmed that down-regulation of KLK6 results in increased endogenous α-syn [16]. Like CAPN1, KLK6 cleaves monomeric α-syn within the NAC region, with a major cleavage site between Lys80-Thr81 [17], and is therefore predicted to protect against α-syn aggregation and neurotoxicity by reducing monomeric α-syn level. Although minor cleavage sites have been identified and are predicted to produce C-terminal truncated α-syn fragments [17], there is no evidence to date to suggest that KLK6-mediated α-syn cleavage, unlike that mediated by CAPN1 and other proteases such as cathepsin D [11] and MMP [55], promotes α-syn aggregation. A recent study demonstrated that elevating the level of KLK6 *in vivo* protected against α-syn aggregation and toxicity in a rat model of PD [25] and that KLK6 level was reduced in human DLB post-mortem brain. KLK6 level is also reduced in CSF in patients with synucleinopathy [56]. As is the case for CAPN1, the phosphorylated and mutant forms of α-syn that accumulate in sporadic and familial Lewy body diseases are more resistant than wild-type unmodified α-syn to cleavage by KLK6 [14,17]. Together, these data provide a strong case for a regulatory role for KLK6 and suggest that reduced KLK6-mediated cleavage of α-syn and α-syn-P129 contribute to the pathogenesis of Lewy body diseases.

As with CAPN1 several outstanding questions remain. Firstly, it has been established that KLK6 is synthesised as an inactive zymogen [20] and secreted extracellularly, whereupon it is activated and degrades extracellular α-syn [22]. This may be particularly important in preventing the transcellular spread of α-syn that is thought to be important in the propagation of disease [23,24]. It is unclear whether intracellular KLK6 is enzymatically active; however, a previous study showed that under stress conditions KLK6 is released from mitochondria into the cytoplasm, where it can interact with and accelerate α-syn degradation [16]. Our finding that KLK6 is partially co-expressed with α-syn at the plasma membrane requires confirmation but would be in keeping with a role for KLK6 in the degradation of α-syn immediately after endocytosis. Lastly, we have used a sandwich ELISA to measure KLK6 protein level in brain tissue samples and have used these measurements as a proxy measure for KLK6 enzyme activity. This approach, however, requires further validation as it is not always the case that protein levels accurately reflect enzyme activity. Dysregulation of KLK6 in DLB by mechanisms acting independently of α-syn may also impact on disease progression. Recent findings suggest that KLK6 indirectly mediates proteolysis of α-syn by regulating the activation of an unidentified metalloprotease [57].

Together, our findings support previous studies highlighting a potential regulatory role of CAPN1 and KLK6 in the metabolism of α-syn and suggest a role for reduced CAPN1 and KLK6 in the pathogenesis of DLB.

## Additional file

Additional file 1: Table S1 MRC UK Brain Bank Network identifiers.
