# Supplementary material for: Accumulation of α-synuclein in dementia with Lewy bodies is associated with decline in the α-synuclein-degrading enzymes kallikrein-6 and calpain-1
Source: Acta Neuropathol Commun. 2014 Dec 5;2:164. doi: 10.1186/s40478-014-0164-0 (PMC4271448; doi:10.1186/s40478-014-0164-0)
Supplement: Additional file 1: Table S1 — MRC UK Brain Bank Network identifiers. [file 40478_2014_164_MOESM1_ESM.docx]

| BBN_8700  BBN_8723  BBN_8759  BBN_8779  BBN_8980  BBN_8983  BBN_9028  BBN_9292  BBN_9299  BBN_9311  BBN_9329  BBN_9340  BBN_9344  BBN_4205  BBN_9354  BBN_9359  BBN_4229  BBN_9365  BBN_9389  BBN_8923 | BBN_9005  BBN_9163  BBN_9164  BBN_9173  BBN_9181  BBN_9182  BBN_9189  BBN_9194  BBN_9262  BBN_9275  BBN_9280  BBN_9303  BBN_4204  BBN_4215  BBN_4216  BBN_9367  BBN_9378  BBN_9189  BBN_9182  BBN_9328 | BBN_9017  BBN_9039  BBN_9064  BBN_9135  BBN_9316  BBN_9321  BBN_9334  BBN_4198  BBN_9351  BBN_9370  BBN_9384  BBN_9032 |
| --- | --- | --- |

**Additional file 1: Table S1** MRC UK Brain Bank Network identifiers.
